# Macrostructures and rhetorical moves in research articles in nanotechnology

**DOI:** 10.3389/fpsyg.2025.1468614

**Published:** 2025-01-22

**Authors:** Xianqing Luo, Jiajie Ji

**Affiliations:** ^1^School of Foreign Languages, Jiangsu University of Technology, Changzhou, China; ^2^School of Foreign Languages, Yangzhou University, Yangzhou, China

**Keywords:** macrostructure, genre analysis, rhetorical moves, nanotechnology RAs, pedagogical implications

## Abstract

**Introduction:**

Notwithstanding the fact that extensive studies focused on exploring generic structures in established disciplines, scant attention has been directed to macrostructures and rhetorical moves in RAs in some emerging disciplines.

**Methods:**

Based on a self-compiled corpus of 50 research articles (RAs) from five top ranking journals in the field of Nanotechnology, we explored the macrostructures and rhetorical moves in RAs in Nanotechnology.

**Results:**

It was found that (i) scientists in Nanotechnology have a propensity to employ a merged [R&D] structure to immediately discuss and contribute new knowledge tentatively in the specific contextualised situation after research results were presented, (ii) Even though RAs in Nanotechnology largely follows IMRD structure by abiding writing conventions in the research world, disciplinary variations were found regarding the rhetorical structures, specifically, scientists in Nanotechnology tend to employ more moves and steps in establishing research niche, detailed description of research methods, suggesting future research, but less moves and steps related to promotional strategies (M3S4, M3S5, M3S6).

**Discussion:**

The research results have significant implications for English for Academic Purposes (EAP) instructors to make informed choices by using disciplinary empirical-based decisions to guide novice writers and students at tertiary level to write RAs by following disciplinary conventions of research communities in Nanotechnology so as to avoid overgeneralization.

## 1 Introduction

It is universally acknowledged that research articles (RAs) are considered the “central genre of knowledge production” and “the main means by which the majority of academics disseminate their work and establish their reputations” (Hyland, 2009, p. 107). Researchers and postgraduate students globally are required to write and publish research articles in reputable journals to advance their careers and enhance their international visibility. Nevertheless, despite the pivotal role of research articles in the dissemination of new knowledge, novice scholars across various disciplines often face challenges in writing research reports for submission to high-impact international journals in English (e.g., Martín and León Pérez, 2014; Swales, 2004). The challenges become particularly pronounced in contexts where novice writers are tasked with navigating issues pertaining to macrostructural organization and the deployment of rhetorical moves within research articles specific to their respective academic disciplines (Breeze and Dafouz, 2017; Ye, 2019). Therefore, it is essential to develop instructional materials specifically tailored to address the pedagogical needs of novice writers. It is of paramount importance to examine how research pertaining to academic genres can inform instructors in making empirically-based decisions when mentoring novice writers in the construction of the distinct components of a research report.

The macrostructure of a discourse, as conceptualized by van Dijk (1980), serves as a conventional framework for organizing global content, offering a structured pathway for readers to navigate complex information. In the realm of academic writing, the study of research article macrostructures often centers on the Introduction–Method–Results–Discussion (IMRD) framework, a format widely regarded as the standard organizational pattern across many disciplines (Swales, 1990; Hartley, 1999). This format encapsulates the logical progression of scientific inquiry, aligning with the “logical cycle of inductive inquiry” (Bruce, 1984), whereby research is introduced, explained, analyzed, and contextualized within a broader academic discourse. The IMRD structure has been historically associated with disciplines that emphasize empirical investigation, such as Biomedical Sciences, where it was institutionalized in the *British Medical Journal* to reflect standardized reporting practices (Bruce, 1984; Hartley, 1999). Over time, the IMRD format has become an archetypal model, adopted widely across disciplines. However, empirical studies have revealed significant variations in its implementation, reflecting disciplinary norms, methodological preferences, and rhetorical priorities. For instance, in Applied Linguistics, RAs often expand beyond the IMRD framework to include additional sections such as Theoretical Basis, which provides a conceptual underpinning for the study, or Pedagogic Implications, which connects research findings to instructional practices (Yang and Allison, 2003, 2004). Similarly, in Information Systems, unique structural elements like Research Model, Problem Analysis, and Evaluation are commonly integrated, reflecting the field’s emphasis on problem-solving and technological application (Kwan, 2017). These modifications underscore the field-specific needs to adapt macrostructural conventions to accommodate distinct research priorities. Likewise, in Economics, the macrostructural organization often includes specialized sections such as Mechanisms, Application, and Robustness, which emphasize theoretical modeling and practical applicability (Jin et al., 2020). Energy Engineering research articles frequently follow a hybridized Introduction–Literature Review–Method–Results and Discussion–Conclusion (ILMR&DC) structure, enabling a seamless integration of findings and their implications (Ye, 2019). In the Humanities, where argumentative and interpretative methods dominate, sections may emphasize detailed literature reviews or theoretical explorations rather than strict adherence to IMRD conventions (Lin and Evans, 2012).

This diversity in macrostructural patterns reflects the influence of disciplinary conventions on textual organization. While the IMRD framework provides a foundational template for organizing empirical research, disciplines adapt and expand its structure to address their unique methodological and rhetorical demands. These adaptations reveal the dynamic interplay between the stability of macrostructural conventions and the variability introduced by disciplinary needs and practices. Just as Ye (2019, p. 50) argued that “the RA macrostructure of any particular area should not be taken for granted without systematic investigation of a considerably large number of representative texts in this area.” By systematically investigating these structures across disciplines, researchers can uncover the shared and distinctive organizational patterns that shape academic communication. Such insights are not only theoretically significant but also pedagogically valuable, particularly in the design of disciplinary-specific writing instruction for novice writers.

Regarding the rhetorical structures in research articles, the concept of rhetorical moves and steps, rooted in Swales’ (1990) seminal genre analysis framework, offers a detailed lens for understanding how texts achieve their communicative purposes within academic discourse. Swales (1990, p. 58) defines a genre as “a class of communicative events, the members of which share some set of communicative purposes”, and these purposes are realized through a sequence of moves, each serving distinct rhetorical functions. A move operates as a functional unit, guiding the reader toward the writer’s communicative intent, while steps provide specific strategies that elaborate or operationalize the move. This analytical approach has proven especially influential in examining the rhetorical structure of research articles, a genre central to academic communication. Swales’ (1990) Create a Research Space (CARS) model has become a cornerstone of rhetorical move analysis, particularly in the study of RA introductions. This model delineates three primary moves: Move 1: Establishing a Territory, this move introduces the broader field of research by reviewing existing literature, highlighting the topic’s significance, or asserting the centrality of the research area. Strategies often include generalizations about the domain, claims of importance, and references to key studies. Move 2: Establishing a Niche, authors identify gaps in the literature, raise unresolved questions, or challenge prevailing assumptions. This move creates the rhetorical space for the current study by signaling a need for further investigation. Move 3: Occupying the Niche, the final move outlines the objectives, scope, and significance of the study, often coupled with a preview of the article’s structure. This move reaffirms the study’s contribution to the field and situates it within the scholarly conversation. Swales’ original model, while widely adopted, underwent significant revision in 2004 to address the variability observed in empirical studies. In the revised CARS model, steps within each move were reclassified as optional or obligatory, acknowledging the fluidity and adaptability of rhetorical structures across disciplines.

A substantial body of studies have validated and extended Swales’ framework, demonstrating its applicability across diverse disciplines and sections of RAs, such as Conservation Biology (Samraj, 2002), Computer Science (Shehzad, 2008, 2010), Agricultural Science (Del Saz-Rubio, 2011), Forestry (Joseph and Lim, 2018, 2019), Mechanical Engineering (Le and Pham, 2020), Educational Psychology (Loi and Evans, 2010), Applied Linguistics (Hirano, 2009; Lim et al., 2014; Ozturk, 2007), Ethnic Studies (Lim and Luo, 2020; Luo and Lim, 2021, 2022), Industrial Relations (Lim and Luo, 2020; Luo and Lim, 2021, 2022), Material Science and Agricultural Science (Guo and Lim, 2024), Energy Engineering (Ye, 2019), and Information Systems (Kwan, 2017). Previous research has identified disciplinary variations in rhetorical structures and their frequencies of occurrence. Conversely, emerging disciplines such as Nanotechnology have received relatively less scholarly attention. Consequently, we have chosen to concentrate exclusively on Nanotechnology to provide essential insights that can inform future genre-based research.

Despite the extensive research conducted on major moves and steps in Introduction (e.g., Lim and Luo, 2020; Luo and Lim, 2021, 2022; Moghaddasi and Graves, 2017; Ozturk, 2007; Del Saz-Rubio, 2011), Methods (e.g., Bruce, 2008; Riazi et al., 2020; Lim, 2006, 2019; Martínez, 2003), Results (e.g., Brett, 1994; Bruce, 2009; Kwan and Chan, 2014; Williams, 1999), Discussion (e.g., Basturkmen, 2012; Geng and Wharton, 2016; Hopkins and Dudley-Evans, 1988; Liu and Buckingham, 2018; Sadeghi and Alinasab, 2020), Conclusion (e.g., Bunton, 2005; Loi et al., 2016) and the entire RAs (e.g., Kanoksilapatham, 2015; Tessuto, 2015; Ye, 2019), a comprehensive examination of the entire rhetorical structures within some burgeoning fields remain absent. Among these different sections, an essential component of research articles that warrants careful examination is the Results section. Although considered a critical part of the manuscript, as it “drives the paper and should therefore be written first” (Cargill and O’Connor, 2006, p. 210), its construction remains challenging. To address this, existing studies have provided insights into its rhetorical structures, particularly highlighting variations across disciplines. Weissberg and Buker (1990), in their seminal textbook *Writing up Research*, proposed a foundational model for structuring the Results section based on cross-disciplinary analyses. This model identifies three key rhetorical elements: (1) locating the figures or tables presenting the results, (2) presenting primary findings categorized into group comparisons, temporal trends, or variable relationships, and (3) commenting on the findings, which include generalizations, explanations, or comparisons to previous studies. These comments commonly utilize linguistic features such as past tense, modal verbs, and hedging. Weissberg and Buker further observed two predominant organizational patterns: (a) Result → Comment and (b) Result → Result → Comment. However, their model, while foundational, has been critiqued for oversimplifying the diverse rhetorical strategies employed in academic writing. Subsequent research has extended this foundational framework. Kanoksilapatham (2005) identified four distinct rhetorical moves within Results sections: reporting results, commenting on results, discussing methodology-related issues, and consolidating findings. The analysis revealed that while “reporting results” was an obligatory move, the inclusion of commentary and methodological reflections varied depending on the article’s purpose. Kanoksilapatham (2015) further refined these findings through a study of engineering sub-disciplines, demonstrating discipline-specific preferences. For instance, biomedical engineering emphasized explanatory commentary, whereas software engineering prioritized concise reporting. These distinctions underscore the importance of tailoring pedagogical approaches to the rhetorical norms of specific fields. Similarly, Ye (2019) analyzed the practices of Chinese authors in energy engineering and observed a strong adherence to international conventions, particularly the IMRD macrostructure. Certain moves, such as “interpreting significance,” were identified as quasi-obligatory. These findings suggest that explicit instruction in move-step structures could help novice writers navigate common barriers to publication. Other discipline-specific analyses provide further insights. Thompson (1993) found that biochemistry Results sections often include evaluative remarks, such as agreement with prior studies (38%) and calls for further research (19%). Brett (1994) reported that 30% of the sociological Results sections in his corpus contained comments, while Williams (1999) observed that 50% of medical Results sections included commentary. Posteguillo (1999) identified that “comparison of findings with literature,” a specific form of commentary, appeared in 50% of computing Results sections. Tessuto (2015) focused on empirical law research articles, reporting that 100% included key findings linked to tables or figures, 100% incorporated commentary, and 71% restated data collection and analysis procedures. These studies highlight the heterogeneity of rhetorical moves in Results sections across disciplines. While “reporting results” remains a core element, the extent of commentary and broader contextualization varies significantly, reflecting discipline-specific communicative goals. These insights lay a robust foundation for the development of targeted instructional materials aimed at improving academic writing proficiency across diverse fields.

The body of research on rhetorical moves and steps highlights their centrality to understanding academic genres as reflections of disciplinary and professional cultures (Bhatia, 2008). As Bazerman (1988) and Berkenkotter and Huckin (1995) observe, genres exhibit a dynamic interplay between stability and change, shaped by evolving disciplinary practices. This duality is particularly evident in the production of RAs, where established members of discourse communities leverage conventional moves while innovating to meet emerging communicative needs (Swales, 1990; Bhatia, 2004). Rhetorical move analysis offers a robust framework for deconstructing the communicative strategies of RAs. For EAP practitioners, these findings underscore the importance of integrating rhetorical move analysis into instructional materials. Studies like Stoller and Robinson (2013) advocate for pedagogical application to enhance students’ genre awareness. By focusing on both the conventional and discipline-specific aspects of rhetorical moves, EAP instruction can better equip students to navigate the complexities of academic writing.

Despite an extensive body of genre-based research, there remains uncertainty regarding (1) what macrostructures and rhetorical moves are employed and (2) to what extent they occur in research articles in some major emerging disciplines (Swales, 2019). This study, therefore, addresses this gap by investigating the macrostructures and rhetorical moves of research articles, as well as analyzing the frequency of rhetorical moves in Nanotechnology, in response to Swales’ (2019, p. 81) call for further exploration. Such an inquiry compels us to evaluate whether notable disciplinary frequency variations exist within emerging disciplines. Such information is crucial for enabling instructors to make well-informed decisions when guiding novice writers in the presentation of RAs within these disciplines, which have been comparatively underexplored in prior research. Actually, few have focused on the macrostructures and rhetorical moves in the field of Nanotechnology, which is a burgeoning field that promises revolutionary advancements across various sectors, including medicine, electronics, and materials science.

Our in-depth focus of this study was primarily driven by the necessity to address challenges in determining (i) what prominent macro-structural patterns could be identified in research articles in the field of Nanotechnology, (ii) what rhetorical structures are employed by expert writers in these RAs to realize their rhetorical functions, (iii) to what extent the moves and steps are employed in these RAs. In this study, we have opted to examine rhetorical structures within the field of Nanotechnology with the purpose to investigate disciplinary rhetorical variations of an emerging discipline.

The provided insights into disciplinary variation are instrumental in aiding authors to avoid overgeneralizations when composing research articles within emergent fields. Indeed, the pedagogical practice of research writing instruction frequently necessitates offering guidance to students from diverse disciplinary backgrounds (Moghaddasi and Graves, 2017). It is incumbent upon educators to critically assess the degree to which the findings regarding information elements are (i) restrictively applicable to certain “disciplines from which the genre samples are collected,” or (ii) “more universally applicable to students across the disciplines” concerned (Cheng, 2019, p. 44). In line with the rationale explained above, three research questions are formulated as follows:

1. What prominent macro-structural patterns could be tentatively identified in RAs in Nanotechnology?
2. What are the moves and steps in these RAs to realize their respective rhetorical functions?
3. To what extent do expert writers include these moves and steps in RAs in Nanotechnology?

The first and third research questions aimed to procure quantitative, text-based data concerning the prevalence of macrostructures and rhetorical structures, as well as the proportion of texts encompassing each specific move and step within this emerging field. Conversely, the second research question focused on obtaining qualitative, text-based data regarding the moves and steps utilized by expert authors in this discipline.

## 2 Research methods

The ensuing sections provide a detailed account of (i) the methodology employed in the compilation of our research article corpus within the field of Nanotechnology for the purposes of this study, and (ii) the procedures utilized in the coding process to assess and ensure percentage reliability.

This study employed two widely recognized text selection methods to construct a research corpus tailored to the objectives of the investigation. First, the content of the selected papers was meticulously examined to ensure the journals represented the field of nanotechnology accurately. Disciplinary experts were consulted in journal selection so that the insights from the study could be “more relevant and acceptable to the disciplinary communities” (Bhatia, 2008, p. 166). Second, the study prioritized top-ranking, highly prestigious journals within the field, as evidenced by their impact values in the *Web of Science* database (e.g., Kanoksilapatham, 2005, 2015; Lim, 2012, 2019; Nwogu, 1997; Posteguillo, 1999; Stoller and Robinson, 2013; Ye, 2019). In line with these criteria, a corpus comprising 50 research articles was compiled from five leading journals in nanotechnology: *Nano Today*, *Nanotechnology*, *Nano-Micro Letters*, *Journal of Nanobiotechnology*, and *International Journal of Nanomedicine*. Specifically, 10 most recently published empirical research articles (Swales, 1990) were randomly selected from each journal, spanning 2 years (2022–2023) at the start of the study. These articles, referred to as “new data” (Swales, 1990, p. 161), were chosen to reflect the dynamic and evolving nature of academic genres. All selected journals were classified as either Quartile 1 or Quartile 2 according to the *Journal Citation Reports*, with impact factors ranging from 0.631 to 6.484 (Thomson Reuters, 2023), underscoring their leading status within the field of Nanotechnology.

To analyze the rhetorical structures within the aforementioned corpus, we employed the coding protocol established by Moreno and Swales (2018, p. 48). This approach necessitated treating “moves as truly ‘functional’ rather than ‘formal’ units,” enabling us to annotate each text fragment at both the move and step levels based on their communicative functions. Consistent with the recommendations of Biber et al. (2007), coding was performed by two independent coders. The primary unit of analysis was the rhetorical step, which could be minimally realized by a clause or more typically by a sentence, a group of sentences, or even several paragraphs, provided it performed a communicative function corresponding to the functional label assigned to the step.

The reliability of the aforementioned coding was assessed using the metric of “percent agreement,” which denotes “the number of agreements per total number of coding decisions” (Biber et al., 2007, p. 35). The “number of agreements” is defined as the instances where the coding decisions made by the first and second coders were identical for each corpus. The coding agreements achieved in this study are presented as follows:

As indicated above, the inter-coder agreement in the first round was 96.37%, thereby exceeding the criteria for inter-coder reliability, which should be above 85.0% (Miles et al., 2014). Six months subsequent to the first coding round, the two coders “discussed their segmentation and labeling” (Moreno and Swales, 2018, p. 51) and “the specialist informants were consulted again” (Ye, 2019, p. 52) to resolve the discrepancies arising from the independent coding decisions. Following this discussion, a second round of coding (recoding) was conducted collaboratively by both coders for each segment, ultimately resulting in complete inter-coder agreement.

The classification of a move or step can be determined using two distinct sets of criteria. According to the first set, a move or step is categorized as “obligatory” if it appears in 100% of the texts, “quasi-obligatory” or “largely stable” if it appears in 51–99% of the texts, and “optional” if it appears in less than 50% of the texts (Lim, 2014, p. 70). The second set of criteria classifies a move or step as “obligatory” if it appears in 100% of the texts, “conventional” if it appears in 60–99% of the texts, and “optional” if it appears in less than 60% of the texts (Kanoksilapatham, 2015, p. 78). However, the latter criteria introduce ambiguity, as a move deemed “obligatory” could also be interpreted as “conventional.” Consequently, this paper adopts Lim’s (2014) criteria, wherein the distinctions between “obligatory” and “quasi-obligatory” are more clearly delineated.

## 3 Results and discussion

### 3.1 Macrostructure patterns

As illustrated in Table 1, the Introduction (I) section is the only segment present in 100% of the research articles (RAs) within the field of Nanotechnology. Notably, the Methods (M) section reveals a pronounced tendency among authors to utilize a “Material and Methods” (Mm) subsection, which appears in over half (54%) of the RAs. Furthermore, a significant proportion (72%) of these articles integrate the Results and Discussion (RD) sections into a single unit, as opposed to presenting them as distinct Results (R) and Discussion (D) sections, which occur in 26 and 22% of the articles, respectively. Additionally, the Conclusion (C) section is a prominent feature, appearing in 94% of the RAs. In addition, Table 2 shows that the exact IMRD is found in only 4% of RAs in Nanotechnology, such finding is consistent with Ye’s (2019) finding that no exact IMRD was found in RAs in energy engineering and supports idea that the exact IMRD structure only employed in limited disciplines (Lin and Evans, 2012). A further analysis shows that variations of IMRD structures were found in an overwhelming majority of RAs. Specifically, the most popular structure in RAs in Nanotechnology is IMR&DC (58%), with a merged R&D section, followed by IMR&DCS (14%) and IMRDC (14%). Such findings reveal that RAs in Nanotechnology predominantly adhere to the IMRD structure as delineated by Swales (1990, 2004). However, notable variations of this structure are observed, with the most prominent being a combined Results and Discussion ([RD]) section in 72% of the articles, as opposed to separate Results (26%) and Discussion (22%) sections. This finding resembles Guo and Lim’s (2024) finding that nearly three-quarters (74%) of RAs in Material Science incorporate a merged [RD] section, but still lower than that in Energy Engineering (88%) (Ye, 2019). Such tendency of employing a merged [RD] can be attributed to the necessity of interpreting and explaining a series of experimental results immediately after their presentation (Ye, 2019), as the Results section presents factual data while the Discussion section addresses interpretative “points” (Swales and Feak, 2004, p. 269). Thus, it can be inferred that scientists in Nanotechnology typically integrate their newly obtained results with their commentary during the process of “generating new knowledge” (Guo and Lim, 2024, p. 6). It is generally recognized that results are presented with a higher degree of certainty, whereas comments often exhibit greater tentativeness (Brett, 1994; Guo and Lim, 2024; Lim, 2010; Weissberg and Buker, 1990). Furthermore, the merged [RD] section is perceived as reader-friendly and demonstrates “writer responsibility” (Soltani and Kuhi, 2022, p. 1) by assisting readers in comprehending new results within the context of the authors’ explanations and interpretations. Therefore, it is understandable that scientists in Nanotechnology tend to support their definite results with tentative comments in the merged RD section, thereby providing readers with meaningful explanatory and contextual information. Another significant structural feature is the Conclusion (C) section, which appears in 94% of RAs and is intended to summarize the major research findings, related discussion points, and pedagogical implications (Lin and Evans, 2012). Consequently, it is understandable that the most prevalent structural pattern in RAs in Nanotechnology is IMR&DC (58%), followed by IMR&D (14%), IMRDC (14%), and other patterns.

**Table 1:** Distribution of section headings (*n* = 50).

RA number							
	NT	N	NL	JN	IN	Total	Percentage
Heading	*n* = 10	*n* = 10	*n* = 10	*n* = 10	*n* = 10	*n* = 50	100
I	10	10	10	10	10	50	100
M	9	10	10	10	10	49	98
E	2	5	9	0	1	17	34
Mm	5	4	1	8	9	27	54
M	2	1	0	2	0	5	10
R	4	2	0	3	4	13	26
D	4	1	0	2	4	11	22
[RD]	6	7	10	7	6	36	72
[DC]	0	0	0	1	0	1	2
C	9	9	10	9	10	47	94
S	0	0	0	9	0	9	18

I, Introduction; E, Experimental; Mm, Material and methods; M, methods; R, Results (separate); D, Discussion (separate); [RD], Results & Discussion (merged); C, Conclusion S, Supplementary information; NT, Nano Today; N, Nanotechnology; NL, Nano-Micro Letters; JN, Journal of Nanobiotechnology; IN, International Journal of Nanomedicine.

**Table 2:** Distribution of various macrostructures (*n* = 50).

RA number							
Pattern	NT	N	NL	JN	IN	Total	Percentage
IMRDC	3	0	0	0	4	7	14
IMR&DC	5	8	10	0	6	29	58
IMRD	1	1	0	0	0	2	4
IMRC	0	1	0	0	0	1	2
IMR&DCS	0	0	0	7	0	7	14
IMRDCS	0	0	0	2	0	2	4
IMRD&CS	0	0	0	1	0	1	2
IR&DC	1	0	0	0	0	1	2

### 3.2 The rhetorical moves and steps

Table 3 presents the results of the move-step analysis, including (1) the moves and steps found in RAs in Nanotechnology, (2) frequencies of RAs including each move and step, and (3) status of each move and step. The subsequent subsections provide detailed explanations of the identified moves and steps, illustrated with examples. Additionally, explicit linguistic signals associated with each move and step are underlined for clarity.

**Table 3:** Distribution of rhetorical moves and steps (*n* = 50).

Moves and steps			No. of RAs including move/step	Percentage	Status
Introduction					
M1	Establishing a territory		50	100	Obligatory
	M1S1	Claiming centrality	50	100	Obligatory
	M1S2	Making topic generalization(s)	50	100	Obligatory
	M1S3	Reviewing items of previous research	50	100	Obligatory
M2	Establishing a niche		48	96	Quasi-obligatory
	M2S1A	Indicating a gap	46	92	Quasi-obligatory
	M2S1B	Adding to what is known	40	80	Quasi-obligatory
	M2S2	Presenting positive justification	39	78	Quasi-obligatory
M3	Presenting the present work		50	100	Obligatory
	M3S1	Announcing present research descriptively and/or purposively	50	100	Obligatory
	M3S2	Presenting RQs or hypotheses	39	78	Quasi-obligatory
	M3S3	Definitional clarifications	24	48	Optional
	M3S4	Summarizing methods	33	66	Quasi-obligatory
	M3S5	Announcing principal outcomes	31	62	Quasi-obligatory
	M3S6	Stating the value of the present research	26	52	Quasi-obligatory
	M3S7	Outlining the structure of the paper	23	46	Optional
Methods					
M4	Developing a theoretical model		43	86	Quasi-obligatory
	M4S1	Explaining scientific principles or mechanisms	31	62	Quasi-obligatory
	M4S2	Deriving mathematical equations	21	42	Optional
M5	Describing experiments		47	94	Quasi-obligatory
	M5S1	Describing sample preparation procedure	47	94	Quasi-obligatory
	M5S2	Describing experimental setup or equipment	47	94	Quasi-obligatory
	M5S3	Describing experimental procedure	46	92	Quasi-obligatory
	M5S4	Considering ethical standards	30	60	Quasi-obligatory
M6	Describing data analysis procedure		46	92	Quasi-obligatory
	M6S1	Providing data sources	46	92	Quasi-obligatory
	M6S2	Describing numerical analysis method	46	92	Quasi-obligatory
	M6S3	Briefing performance assessment method	28	56	Quasi-obligatory
Results and discussion					
M7	Reporting results		50	100	Obligatory
	M7S1	Restating research purposes and procedures	46	92	Quasi-obligatory
	M7S2	Restating research hypothesis	39	78	Quasi-obligatory
	M7S3	Presenting overall experimental or analytical results	50	100	Obligatory
	M7S4	Highlighting specific significant results	50	100	Obligatory
	M7S5	Making generalizations about the results	50	100	Obligatory
M8	Commenting on the results		50	100	Obligatory
	M8S1	Interpreting the results	50	100	Obligatory
	M8S2	Providing a reason for the results	50	100	Obligatory
	M8S3	Comparing the results with previous studies	50	100	Obligatory
	M8S4	Explaining possible errors or pointing out limitations	27	54	Quasi-obligatory
M9	Evaluating the results		42	84	Quasi-obligatory
	M9S1	Stating the advantages of the results	40	80	Quasi-obligatory
	M9S2	Suggesting the applicability of results	42	84	Quasi-obligatory
	M9S3	Claiming a solution to the problem	21	42	Optional
Conclusion					
M10	Reviewing the present study		50	100	Obligatory
	M10S1	Briefing purposes and methods	50	100	Obligatory
	M10S2	Summarizing results	49	98	Quasi-obligatory
	M10S3	Exemplifying results	39	78	Quasi-obligatory
M11	Evaluating the present study		49	98	Quasi-obligatory
	M11S1	Stating the significance or new insights	49	98	Quasi-obligatory
	M11S2	Suggesting application of the results	49	98	Quasi-obligatory
M12	Promoting future research		45	90	Quasi-obligatory
	M12S1	Stating a practical need for further study	43	86	Quasi-obligatory
	M12S2	Indicating the potential value of further study	44	88	Quasi-obligatory

#### 3.2.1 The moves and steps in the introduction

Move 1 and Move 3 are obligatory moves (100%) in RAs in Nanotechnology, however, Move 2 was found to be a quasi-obligatory move employed in 96% of nanotechnology RAs. The occurrence of Move 1 (M1) in 100% of the texts aligns with findings reported in several other studies, such as those by Samraj (2002) on two environment-related disciplines, Kanoksilapatham (2005) in Biochemistry, Kanoksilapatham (2015) in Civil, Software, and Biomedical Engineering disciplines, and Ye (2019) in Energy Engineering. In M1, authors typically employ rhetorical strategies to facilitate the reader’s comprehension of the research background through following steps: claiming centrality, making topic generalization(s), and reviewing items of previous research. These steps are exemplified in Example (1), (2) and (3) below.

1. [M1S1] Antibacterial surfaces are essential to reduce infections and illness in a wide variety of applications such as medical devices … Surfaces that are antiviral are also of much interest for these applications. The most prevalent antibacterial strategy employs antimicrobials such as organic compounds like triclosan and zinc pyrithione or metallic ions … (RA 11, p. 1)
2. [M1S2] Carbon nanoparticles are a promising alternative to semiconductor nanocrystals as next generation green nanomaterials … (RA 12, p. 1)
3. [M1S3] Sargent et al. [15] demonstrated that BiOBr-templated catalyst preferentially exposed high CO2RR activity Bi (110) facets … (RA 29, p. 2)

Move 2 (M2) encompasses “Step 1A Indicating a gap,” including four sub-steps: “Highlighting the complete absence of research bearing a specific characteristic” “Stressing insufficient research in a specific aspect” “Revealing a limitation in previous research” “Contrasting conflicting previous research findings” (Lim, 2012), “Step 1B: adding to what is known” (Swales, 1990), and “presenting positive justification” (Samraj, 2002; Swales, 2004). Illustrative examples of these steps are provided in Examples (5) and (6).

1. [M2S1A] Such designs, although well demonstrated in probabilistic computing applications, still have essential limitations: (i) the data retention time (i.e., dwell time) is determined by the energy barrier… (ii) Additionally, it is difficult to fabricate a chip with a tight statistical distribution around a small energy barrier for all devices… (iii) A feedback loop is needed for each MTJ to … (RA 13, p. 2)
2. [M2S1B] In this paper, as a complementary approach we report on a detailed study of the influence of oxygen and water on the electrical conductivity of GaN NFs … (RA 16, p. 2)
3. [M2S2] This approach offers several advantages: it can be applied to industry-standard perpendicular MTJs and allows the p-bits to be updated with a high speed and on-demand, comparable or faster than p-bits based on superparamagnetic in-plane MTJs. Furthermore, … (RA 13, p. 2)

Move 2 was found to be a quasi-obligatory move appeared in 96% of Nanotechnology RAs, such frequency aligns with the findings of Samraj (2002) and Ye (2019), but significantly exceeds the results reported by Kanoksilapatham (2015), which ranged from 71 to 81%. In Move 2, “M2S1A indicating a gap” is the most frequently employed step (92%). This frequency is significantly higher than that in Civil Engineering (71.67%), Soft Engineering (81.67%), Biomedical Engineering (86.44%) (Kanoksilapatham, 2015), and Law (69%) (Tessuto, 2015). This disparity can be attributed to nanotechnology being an emerging and rapidly expanding discipline, characterized by numerous research gaps. Scientists in Nanotechnology carefully situate their studies in the research field (Kawase, 2021) and paves the way for new knowledge claims (Soler-Monreal, 2015) via indicating research gaps. In addition, the uncommon step of “presenting positive justification,” identified in the data of Samraj (2002) and later deemed optional by Swales (2004), was observed in 41.18% of Biomedical Engineering research articles by Kanoksilapatham (2015), however, in this study, it occurs with a frequency as high as 68%.Such positive justification serves as an “additional” and “non-face-threatening” strategy, enabling authors to “directly state the value,” “merits,” “the need,” or other “positive reasons for conducting a study” (Samraj, 2002, p. 9).

Furthermore, salient linguistic features can be identified, including discourse markers such as “however” and “thus” to indicate contrast or implication. Lexical choices such as “lack,” “drawbacks,” and “urgent need” highlight gaps, limitations, and research necessities. Phrases like “in order to” and “to overcome” set the context for subsequent actions or needs. Emphatic expressions such as “still needs further investigation” and “has not yet been found” underscore the importance and necessity of continued research. These instances exhibit distinct linguistic features that align with the rhetorical functions of Move 2 (M2) and its associated steps. Collectively, these features contribute to the rhetorical effectiveness of Move 2, emphasizing gaps, limitations, and the necessity for further research or development within the field.

Regarding the seven steps in M3, M3S1 is the only obligatory step, and all the other steps are either quasi-obligatory or optional steps, examples given below, indicating that writers in Nanotechnology can use the rhetorical strategies flexibly.

1. [M3S1] In this work, we map both LSPR modes (dipolar and higher order polarities) and VP modes in nanorod clusters (hexamers and tetramers) fabricated by electron-beam lithography (EBL)… (RA 17, p. 2)
2. [M3S2] What fundamental physico-chemical processes occur when the printed NP ink traces are subjected to simple heating-based sintering? This has been an important question that needs to be answered … (RA 14, p. 2)
3. [M3S3] Zeolitic imidazolate framework-8 (ZIF-8), a typical type of metal–organic frameworks (MOFs) with zeolite topology, is considered as a biocompatible nanobot matrix, … (RA 25, p. 2)
4. [M3S4] In the same stream, the present paper proposes an alternative methodology to measure both the thermal diffusivity and conductivity of GNP strips, … (RA 15, p. 2)
5. [M3S5] The results showed that GA-nCeO2 hydrogel scaffold exhibited a typical three-dimensional porous structure with a mean pore ratio of 70.61 ± 1.94%. (RA 48, p. 6561)
6. [M3S6] Our study brings knowledge for the potential application of nanostructured biomaterials to work as an integrative platform under the detrimental metabolic status present in diabetic conditions. (RA 50, p. 1)
7. [M3S7] The paper is organized as follows. The methodology is detailed in section 2, … (RA 15, pp. 2–3)

In Move 3, the most frequently occurring step is M3S1 (100%), followed by M3S2 (78%), M3S4 (66%), M3S5 (62%), M3S6 (52%), M3S3 (48%), and M3S7 (46%). The frequency of M3S2 in Nanotechnology is notably higher than in Industrial Relations (56.7%) and Ethnic Studies (43.3%) (Lim and Luo, 2020), and Law (20%) (Tessuto, 2015), indicating a propensity among Nanotechnology scientists to provide research questions and/or hypotheses to guide both writers and readers. The frequency of M3S4 (66%) in this study is comparable to Civil Engineering (66.67%), higher than Ethnic Studies (46.67%) (Luo and Lim, 2022) and Law (61%) (Tessuto, 2015), but lower than those in Energy Engineering (92%) (Ye, 2019), Soft Engineering (91.67%), Biomedical Engineering (79.67%) (Kanoksilapatham, 2015), and Industrial Relations (76.67%) (Luo and Lim, 2022). M3S4, M3S5, and M3S6 can be viewed as promotional strategies within research articles to succinctly present novel research methods, results, and contributions to knowledge, thus capturing the interest of readers and reviewers. The data suggests that Nanotechnology scientists need to focus more on including these three steps (M3S4, M3S5, M3S6) in their RAs compared to other disciplines.

#### 3.2.2 The moves and steps in the methods

Among the three moves in the Methods section, Move 4 (developing a theoretical model) and Move 5 (describing experiments) are classified as quasi-obligatory. M4 incorporates two steps: M4S1 explaining scientific principles or mechanisms and M4S2 deriving mathematical equations.

1. [M4S1] Physical damage mainly contributes from its large surface area and sharp edges. The large graphene nanosheet would entrap the bacteria to prevent the nutrient supply to starve the bacteria, while the sharp edges would penetrate the bacterial membranes to extract phospholipid molecules destructively, and then damage RNA to exert antimicrobial effect. (RA 45, p. 4508).
2. [M4S2] Cell viability was calculated according to the following equation. (RA 31, p. 3)

M5, describing experiments, takes three steps: describing sample preparation procedure (M5S1) shown in Example (16), describing experimental setup or equipment (M5S2) shown in Example (17), describing experimental procedure (M5S3) in Example (18), and considering ethical standards (M5S4) in Example (19) below.

1. [M5S1] Ferric chloride (FeCl3) and TA were obtained from Sigma Chemical Co., Ltd. (Saint Louis, MO, USA) … N-hydroxysuccinimide (NHS) and triptolide were from Sinopharm Chemical Reagent Co., Ltd. (Shanghai, China). (RA 35, p. 3).
2. [M5S2] The custom-built Kelvin probe setup is equipped with a commercial controller (Kelvin Control 07, Besocke DeltaPhi) and a piezoelectrically driven gold grid with a diameter of 3 mm and a work function (WF) of 4.9 eV as a probe. (RA 16, p. 3)
3. [M5S3] After centrifugation three times for washing, then the cells were dissolved into the binding buffer. Annexin V-FITC and PI were added (BioVision, Milpitas, CA, United States), and the cells were incubated for 10 min at room temperature in the dark. (RA 31, p. 3)
4. [M5S4] The human subject protocol was approved by the Biomedical Research Ethics Committee of Shandong Provincial Hospital. … (RA 43, p. 5945)

Interestingly, M6, describing data analysis procedure, is also an obligatory move in this study, Nanotechnology RAs need to provide data sources (M6S1) as in Example (20), describing numerical analysis methods (M6S2) as in Example (21), and Briefing performance assessment method (M6S3) as in Example (22).

1. [M6S1] Dimethylsulfoxide (DMSO) and tetrahydrofuran (THF) were purchased from China National Medicines Co. Ltd. (RA 47, p. 6608)
2. [M6S2] SPSS 19.0 and GraphPad 9.0 were used for statistical analyses. All data were presented as mean ± standard deviation (SD). One-way analysis of variance (ANOVA) was used for comparisons of multiple groups. A t-test was followed for comparison between two groups, and the statistical significance was defined as * *p* < 0.05. (RA 1, p. 19)
3. [M6S3] CPD measurements were performed in the same measurement chamber on commercially available not intentionally doped free standing bulk m-plane GaN plates (MSE Supplies LLC) and … (RA 16, p. 3)

In the Methods section, the study found that Moves 4, 5, and 6 (M4, M5, M6) are quasi-obligatory, which occurs in 86, 94, and 92% of RAs in Nanotechnology. The frequency of occurrence of M4 (86%) is higher than that in Energy Engineering (51%) (Ye, 2019). Interestingly, the frequency of occurrence of M5 (94%) in Nanotechnology RAs is slightly lower than the frequency (100%) in the studies of Kanoksilapatham (2005, 2015), Maswana et al. (2015), Nwogu (1997), Stoller and Robinson (2013), but much higher than the frequency (84%) in Ye (2019). The frequency of occurrence of M6 (92%) is slightly lower than that in Energy Engineering (100%) (Ye, 2019). A further analysis indicates that the total frequencies of the steps within these moves are higher than in other disciplines, such as those reported by Ye (2019), Kanoksilapatham (2015), and Tessuto (2015), suggesting that Nanotechnology scientists prioritize providing detailed and comprehensive research methods.

#### 3.2.3 The moves and steps in the results and discussion

The presence of Move 7 (Reporting Results) as an obligatory element (100%) in research articles in Nanotechnology aligns with similar findings in various other disciplines, including Medicine (Nwogu, 1997), Civil Engineering, Biomedical Engineering (Kanoksilapatham, 2015), Chemical Engineering (Maswana et al., 2015), and Energy Engineering (Ye, 2019). This move typically commences with a step that reiterates the research objectives and methodologies to the reader (M7S1). Subsequently, the overall experimental and analytical outcomes are presented (M7S2), with specific significant results being emphasized (M7S3), and broad generalizations being drawn (M7S4).

1. [M7S1] To further determine the components of the hybrid interface after reacting, cryo-transmission electron microscopy (cryo-TEM) was used to analyze its crystal structure and electronic information. (RA 26, p. 5)
2. [M7S2] The experiments unravel two key issues. First and foremost, it established that there is polymeric surface coverage … Second, it provided an estimation of the pore fraction in the sintered traces… (RA 17, p. 5)
3. [M7S3] Compared with the control group, a significant reduction (60.1 and 60.2%) in cellular uptake was observed at 4°C and with colchicin, a microtubule-disrupting drug. (RA 31, p. 8)
4. [M7S4] With such a geometry, we see that the conductivity is now dependent on the direction of electric field … (RA 14, p. 7).

Subsequent to Move 7 is Move 8, which entails commenting on the results. This move involves interpreting the meaning or implications of the results (M8S1), providing explanations for the findings (M8S2) based on theoretical frameworks, scientific principles, or established knowledge, and comparing the results with those of previous studies (M8S3). Within Move 8, potential errors or undesirable outcomes are elucidated, and any limitations of the study are acknowledged (M8S4). Examples (27)–(30) serve to illustrate the four steps encompassed within Move 8.

1. [M8S1] These results indicate that GBT has a significant ability in promoting cell proliferation and migration, which favors the wound healing process. (RA 45, p. 4514)
2. [M8S2] These results should be attributed to the facilitated Zn deposition on Sn@Zn foil, as supported by the EIS spectra of Zn–MnO2 batteries based on different anodes (Fig. 6f). (RA 24, p. 8)
3. [M8S3] These results are in accordance with the literature, which reports an oxygen-induced Fermi level pinning for polar GaN surfaces [34] and GaN NWs [20], [35] (RA 16, p. 7)
4. [M8S4] However, we encountered several experimental challenges with the control groups during the course of the study. First, it was difficult to quantify the concentration (or dose) of ginseng and … This made it problematic to directly conduct *in vitro* and *in vivo* experiments … we were not able to anticipate obtaining meaningful results with them… (RA 31, p. 17)

Following the commentary on the results in Move 8, Move 9 involves the evaluation of the results, encompassing three distinct steps: stating the advantages of the findings (M9S1), suggesting their practical applicability (M9S2), and asserting a solution to the research problem (M9S3). This structure is exemplified in Examples 31–33.

1. [M9S1] Therefore, compared with the nanofiber composite membrane, the NCRO enables more excellent long-term sensing stability and durability. (RA 21, p. 13)
2. [M9S2] This work revealed a novel role of NSC-exo on wound healing, which might contribute to the development of novel therapies for skin injury in the future. (RA 49, p. 5993)
3. [M9S3] Therefore, it is urgent to find a solution to overcome this shortcoming, so as to facilitate the better application of white light. (RA 47, p. 6615)

In the Results and Discussion section, Move 7 (reporting results) and 8 (commenting on the results) are obligatory, while Move 9 (evaluating results) is quasi-obligatory. M7S1 (92%) is quasi-obligatory, and M7S3, M7S4, and M7S5 are obligatory. These findings are consistent with previous studies; however, this study discovered that over three-quarters of Nanotechnology scientists tend to restate research hypotheses (M7S2, 78%), such tendency have not been found in other disciplines such as Energy Engineering (Ye, 2019), Civil Engineering, Soft Engineering, and Biomedical Engineering (Kanoksilapatham, 2015).

#### 3.2.4 The moves and steps in the conclusions

The Conclusion sections are generally succinct and concise, typically comprising three moves: a brief review of the study (M10), including briefing research purposes and methods (M10S1), summarizing methods (M10S2) and exemplifying results (M10S3); a concise evaluation of the study (M11) via stating the significance or new sights (M11S1) and suggesting application of the results (M11S2); and an indication or suggestion for future research (M12) through stating a practical need for further study (M12S1) and indicating the potential value of the further study (M12S2). The steps for each move are outlined below.

1. [M10S1] In summary, we have constructed a novel microbiotic nanomedicine Cu2O@ΔSt by anchoring Cu2O NPs on the surface of engineered *Salmonella typhimurium* strain for achieving … (RA 28, p. 19)
2. [M10S2] In summary, vertical 3D NiO nanoflakes and NiMoNH nanopillars have been successfully synthesized to use as electrocatalysts for the anodic GOR and cathodic HER, respectively … The as-assembled electrolyzer exhibits good HER performance and long-term stability. (RA 22, p. 11)
3. [M10S3] More importantly, in vivo infected-wound healing treated by GBT exhibited faster collagen deposition (with almost no scar formation) and accelerated regeneration of skin tissue at the end of 8 days. (RA 45, p. 4517)
4. [M11S1] Nevertheless, the simplicity and versatility of the nanobot-assisted cell recognition and isolation offer a novel tool for diverse biomedical applications, highlighting foreseeable clinical and commercial opportunity. (RA 25, p. 12)
5. [M11S2] This work opens a new avenue for the practical applications in the future hydrogen economy. (RA 22, p. 11)
6. [M12S1] Although the low level of H2O2 used in this study shows minimal impact on the cell viability, future research directions in nanobot formulation could exploit alternative and … (RA 25, p. 12)
7. [M12S2] We anticipate that future studies will delve into the examination of GENs in combination with conventional drugs, explore other applications of GENs, and investigate possible modifications to enhance their targeting ability. (RA 31, p. 17)

Following the detailed evaluation of specific results in the Results and Discussion, the authors make a concerted effort to underscore the overall significance of their research in the Conclusion. The promotion of further research (Move 12) transitions the focus from the current study to future inquiries, either by articulating a practical need (M12S1) or by indicating the potential value of further study (M12S2). The frequencies of the moves in the Conclusion sections exhibit considerable variation, reflecting the authors’ flexible strategy choices to effectively achieve their communicative objectives. Move 10 (reviewing the present study) is obligatory, while Moves 11 (evaluating the present study) and 12 (promoting future research) are quasi-obligatory. The study highlights a notable rhetorical feature: Nanotechnology scientists show a greater propensity to promote future research (M12, 90%) compared to other disciplines, such as Energy Engineering (14%) (Ye, 2019) and Law (31%) (Tessuto, 2015). This trend is likely due to nanotechnology being an emerging and burgeoning field, necessitating recommendations for future research based on current studies.

## 4 Summary

This study investigates the macrostructures and rhetorical moves in Nanotechnology research articles. Addressing the first research question concerning prominent macrostructural patterns, this study found that scientists in Nanotechnology have a propensity to employ a combined Results and Discussion [R&D] structure in their research articles to immediately discuss and contribute new knowledge in the specific contextualised situation after research results were presented. In addition, the Conclusion section is consistently utilized to underscore key findings and implications, with the IMR&DC pattern identified as the most commonly employed macrostructure. Regarding the second research question on the rhetorical moves and steps employed, among the 12 moves identified across the RAs, M1, M3, M7, M8, and M10 are obligatory and M2, M4, M5, M6, M9, M11, and M12 are quasi-obligatory. Among the 40 identified steps, 11 are obligatory, 26 quasi-obligatory, and 3 optional (refer to Table 3), reflecting largely adherence to established genre conventions. However, regarding the third research question formulated for this study, certain rhetorical variations are evident. In the Introduction section, scientists in Nanotechnology normally employ an expanded range of moves and steps to establish the research territory and situate their studies within the existing body of knowledge. This section also includes heightened emphasis on establishing a research niche to highlight the field’s rapid evolution and emergent nature, as well as prioritizing the articulation of research questions and hypotheses, while promotional moves are comparatively infrequent. In the Methods section, detailed and transparent descriptions of research methods are commonly provided to ensure methodological rigor. Within the [R&D] sections, researchers in Nanotechnology have a marked tendency to integrate research results with their interpretations, fostering a cohesive presentation of findings. In the Conclusion sections, future research directions are often delineated, reflecting the dynamic and continuously evolving nature of the Nanotechnology discipline. Overall, the findings demonstrate that while Nanotechnology RAs largely adhere to established genre conventions, the observed structural and rhetorical innovations reflect the discipline’s distinctive communicative needs. This dual adherence to convention and innovation enables scientists to align with international scientific standards while tailoring their writing practices to foster disciplinary advancement and engagement.

## 5 Pedagogical implications

The findings of this research possess profound pedagogical implications for novice writers and students at the tertiary level within the field of Nanotechnology. Primarily, educators are advised to adapt their instructional materials by integrating authentic examples from research articles (RAs) specific to Nanotechnology and incorporating relevant statistical data from studies within this domain. This approach aims to enhance learners’ engagement with the field and mitigate the risk of overgeneralization from other disciplines. Concerning the macrostructures of RAs in Nanotechnology, students are encouraged to proficiently utilize the IMR&DC structure, as identified as the predominant pattern in this study’s findings. Furthermore, educators should instruct students to adopt a merged [R&D] structure, characterized by presenting a tentative, modest, and contextual discussion immediately following the results. This alignment with the conventions of the Nanotechnology research community is imperative. Additionally, instructors should emphasize the importance of effectively highlighting research gaps to appropriately situate their studies within the field. This involves the expectation of a comprehensive and detailed methods section. Moreover, scientists within the domain of Nanotechnology are encouraged to employ more promotional strategies, specifically through the utilization of Moves 3, Steps 4, 5, and 6 (M3S4, M3S5, and M3S6). Given that Nanotechnology is an emerging and rapidly expanding field, learners should be encouraged to provide extensive information on promoting future research, distinguishing their efforts from those in other disciplines. In summary, instructors must make informed pedagogical decisions by guiding learners in adhering to their disciplinary writing conventions. This guidance is crucial for enabling learners to “gradually acquire specialized discourse competencies that allow them to participate as group members” (Hyland, 2009, p. 48) in their respective international research communities. Future research should prioritize the investigation of the prominent linguistic resources utilized by expert writers in the field of Nanotechnology so as to enrich the corpus of disciplinary resources available and enhance the effectiveness of English for Academic Purposes instruction at the tertiary level.

## Data Availability

The raw data supporting the conclusions of this article will be made available by the authors, without undue reservation.
